# Medical Controversies

**Published:** 1847-04

**Authors:** 


*Medical Controversies.*—Dr. Cheyne remarks, in reference to a work which he wrote against a treatise by M. De Moivre, that, "being written in a spirit of levity and resentment, I most sincerely retract and wish undone, so far as it is personal or peevish, and ask him and the world pardon for it, as I do for the defence of Dr. Pitcairn's Dissertation on Fevers against the late learned and ingenious Dr. Oliphant. *I heartily condemn and detest all personal reflections, all malicious and unmanly terms, and all false and unjust representations*, as unbecoming gentlemen scholars and Christians, and disapprove and undo both performances, as far as in me lies, in everything that does not strictly and barely relate to the argument."—*Life of Dr. Cheyne.*

Many modern controversialists of the medical profession might profit by the example of this candid old physician of the seventeenth century. Nothing can more strikingly show the absurdity of indulging in violent controversies on medical subjects, than the utter indifference of another generation to them, and the complete oblivion into which they sink in a course of a few years. But medical writers are of an *irritabile genus;* and it is a rarity to meet with such an example of candour and good feeling as that shown by Dr. Cheyne.—*Lond. Med. Gaz.*

